# Association between pre-treatment IQ and educational achievement after gender-affirming treatment including puberty suppression in transgender adolescents

**DOI:** 10.1177/13591045221091652

**Published:** 2022-05-31

**Authors:** Marijn Arnoldussen, Evelien C Hooijman, Baudewijntje PC Kreukels, Annelou LC de Vries

**Affiliations:** 1 Department of Child and Adolescent Psychiatry, Center of Expertise on Gender Dysphoria, Amsterdam UMC, Vrije Universiteit Amsterdam, Amsterdam, Netherlands; 2 Department of Medical Psychology, Center of Expertise on Gender Dysphoria, Amsterdam UMC, Vrije Universiteit Amsterdam, Amsterdams, Netherlands

**Keywords:** Gender dysphoria, gender incongruence, transgender adolescents, puberty suppression, gender-affirming medical treatment, intelligence, educational achievement

## Abstract

**Background:**

Concerns exist regarding effects of puberty suppression on neurodevelopment. Intelligence is strongly correlated with educational achievement in the general population. This study aimed to examine the association between pre-treatment intelligence and educational achievement after gender-affirming treatment including puberty suppression in transgender adolescents to contribute to the emerging understanding of the effect that gender-affirming treatment including puberty suppression may have on cognitive development.

**Methods:**

IQ was measured in 72 adolescents (45 trans boys, 27 trans girls) at clinical entry (mean age 12.78 years), educational achievement was evaluated after gender-affirming treatment (mean age 20.40 years).

**Results:**

IQ pre-treatment and educational achievement post-treatment were positively associated (Nagelkerke R = 0.71).

**Discussion:**

The association between IQ pre-treatment and educational achievement post-treatment in transgender adolescents who received gender-affirming medical treatment including puberty suppression appears to be similar to the general population. This may reflect that gender-affirming medical treatment including puberty suppression does not negatively affect the association between IQ and educational achievement.

## Introduction

Gender dysphoria (GD) refers to incongruence between a person’s assigned sex based on their biological sex characteristics and their experienced gender, resulting in psychological distress (de Vries et al., 2014; Hembree et al., 2017; Mahfouda et al., 2017).

Puberty suppression (PS) in the form of GnRH analogues may be prescribed in adolescents with GD to delay the development of secondary sex characteristics, providing time for exploring gender identity and relieve the distress of physical pubertal development before any decisions regarding more irreversible steps in gender affirmative treatment are made (de Vries et al., 2014; Mahfouda et al., 2017). PS can be prescribed to transgender adolescents who have shown persistent, long lasting GD, have no interfering psychological difficulties, are socially supported and understand the pros and cons of this treatment (de Vries et al., 2014; Hembree et al., 2017; Mahfouda et al., 2017). The adolescents should have entered puberty Tanner stage G2/B2 to ascertain that they have experienced at least some physical pubertal development. After puberty suppression, transgender adolescents may continue with gender-affirming hormone therapy (GAHT) (Hembree et al., 2017). A study evaluating psychological outcomes in young adulthood of this approach showed improved psychological functioning and general wellbeing comparable to same age peers (de Vries et al., 2014).

Specific information about the impact of PS and GAHT on the maturation of the brain, including their effects on cognitive development, is still limited (Hembree et al., 2017; Richards et al., 2019). Concerns have been raised regarding the risks of PS on neurodevelopment (Chen et al., 2020; Costa et al., 2016; Laidlaw et al., 2019). A study on long-term effects of PS on brain development of sheep who received PS found that long-term spatial memory performance remained reduced after discontinuation of PS (Hough et al., 2017). Another study showed that PS treatment after puberty onset exerts sex-specific effects on social and affective behaviour, stress regulation, and neural activity in mice (Anacker et al., 2021). It is important to emphasize that these results are from research with animal models. The few studies that have been conducted on the effect of PS on cognitive performance in human yielded mixed results. A study in adopted children (*N* = 30) with precocious puberty who were treated with GnRH analogues, either alone or with growth hormone, showed that their IQ levels had decreased about 7 points after this treatment (Mul et al., 2001). However, yet another study found no differences in cognitive performance between 15 GnRHa treated girls with precocious puberty and their age-matched controls (Wojniusz et al., 2016).

Assessing the effect of PS on cognitive development is very challenging. A randomized controlled trial in which some of the adolescents receive PS and others do not, would provide the most accurate estimate of the effect. However, since such studies are not at all desirable from an ethical perspective, other methods will have to be explored to gain a better insight. To what extent IQ at young age and final educational attainment in adulthood are correlated could possibly shed more light on this effect. Numerous studies have shown that cognitive ability, as measured by IQ scores, is positively correlated with educational achievement in the general population (Sternberg et al., 2001). For example, a 5-year prospective longitudinal study in England of over 70.000 children examined the association between intelligence at age eleven and educational achievements at age sixteen and found a strong correlation: 0.81 (Deary et al., 2007). For this reason, we hypothesized that the association between IQ pre-treatment and educational achievement post-treatment in transgender adolescents provides a proxy of the effects that PS followed by GAHT may have on cognitive development.

There are several factors that could potentially affect the association between IQ and educational achievement. Psychological distress as well as behavioural problems are negatively associated with educational achievement (Loe & Feldman, 2007; Rothon et al., 2009). Furthermore, Israel et al. (2001) found that for high school students, the social support of their family is a key factor affecting educational achievement.

This study focused on the association between pre-treatment intelligence (before gender-affirming treatment starting with PS followed by GAHT and affirming surgeries) and post-treatment educational achievement in young adulthood (after gender-affirming treatment) of transgender adolescents. Apart from age and gender, emotional and behavioural problems of the adolescents as well as the family situation and family functioning were examined as covariates in this study.

## Methods

### Participants

This study was performed at the Center of Expertise on Gender Dysphoria (CEGD) of Amsterdam University Medical Centers, location VUmc, Amsterdam, the Netherlands and was part of a larger research project to measure the outcome of early medical intervention in transgender adolescents (de Vries et al., 2014). Adolescents who were referred before 2010, met the criteria for the diagnosis of gender dysphoria (according to the DSM-IV-TR) (American Psychiatric Association, 2000), started with PS before the age of 17 years followed by gender-affirming hormonal treatment and gender-affirming surgery (vaginoplasty, hysterectomy or mastectomy), could be included in this study. There were no exclusion criteria.

Of the 119 adolescents who were eligible for this study, 72 participated. There were several reasons for non-participation; some could not be contacted because correct address information was lacking, some agreed to participate but did not fill out the questionnaires despite repetitive reminders, and some declined to participate. The 72 included subjects were compared on demographic characteristics with the 47 individuals who did not participate in the study. Chi-square tests showed that the sex-ratio was not significantly different between the two groups, but that the included adolescents were significantly more likely to live with their biological parents than the adolescents who did not participate. Independent sample *t*-tests revealed that the included group was significantly younger when they started with puberty suppression. The total IQ and the time between the start with PS and the start with GAHT was comparable between the two groups.

Of the 72 participants, 45 were trans men and 27 were trans women. During data collection, people were not specifically asked if they identified outside the gender binary. The participants received on average 2.40 years (SD 1.08, range 0.52–5.06 years) of PS before starting with GAHT. Demographic characteristics are shown in Table 1.

**Table 1.** Sociodemographic characteristics of the study sample.

Gender, N (%)		
- Trans men	45 (62.5%)	
- Trans women	27 (37.5%)	
Living situation of the adolescent before treatment, N (%)		
- Living with both biological parents	52 (72.2%)	
- Other	19 (26.4%)	
- Unknown	1 (1.4%)	
Age in years	M (SD)	Range
- Clinical entry/baseline	12.78 (1.48)	10.73–16.94
- Start puberty suppression	13.77 (1.46)	11.47–16.99
- Start gender-affirming hormonal treatment	16.22 (0.82)	13.93–18.98
- Gender-affirming surgery	18.70 (0.77)	17.56–21.87
- Evaluation educational achievement	20.40 (1.03)	18.64–23.78
Years between start puberty suppression and start of gender-affirming hormonal treatment	M (SD)	Range
	2.40 (1.08)	0.52–5.06

*Note*. M = mean, SD = standard deviation.

### Procedure

The adolescents followed the usual diagnostic process (de Vries et al., 2014). The participants were assessed two times: pre-treatment (before the start of PS or GAHT, mean age 12.78 years) and post-treatment (after gender-affirming hormones and surgery, mean age 20.40 years). Pre-treatment, sex assigned at birth and the living situation of the adolescent were collected from the medical chart. Depending on age, the IQ was measured using the Wechsler Intelligence Scale for Children (WISC) (Wechsler et al., 2002) or Wechsler Adult Intelligence Scale (WAIS) (Wechsler, 1997), and emotional- and behavioural problems were examined using the total internalizing and externalizing problems scale of the Child Behaviour Checklist (CBCL) (Verhulst et al., 1996), and Youth Self Report (YSR) (Verhulst et al., 1997). Besides, the age at which PS and GAHT were started were collected from the medical charts. Post-treatment assessment took place between 2009 and 2016 and was defined as at least 1 month after affirming surgeries (mastectomy, hysterectomy or vaginoplasty). Participants were invited for research evaluation at the CEDG. Part of this evaluation was a survey about current or finished educational achievement. In the Netherlands, school systems can be divided in pre-vocational, higher pre-vocational and pre-university education (van den Bos et al., 2012). In this study, educational achievement was dichotomized into ‘vocational educated’ and ‘higher vocational educated/academic educated’. ‘Vocational educated’ included pre-vocational education (VMBO in Dutch) and vocational education (MBO in Dutch), depending on the age of the adolescent at the moment of the research evaluation. ‘Higher vocational educated’ included higher pre-vocational education (HAVO in Dutch), pre-university education (VWO in Dutch), higher vocational education (HBO in Dutch) and academic education (university). In the Netherlands, vocational education traditionally focuses on preparing students to work in a trade or craft, while higher vocational and academic education concentrates on higher learning and professional training. Furthermore, family functioning was evaluated post-treatment using the general functioning scale of the Family Assessment Device (FAD) (Epstein et al., 1983).

Informed consent was signed by all adolescents and their parents at the baseline-assessment and by the participants at follow-up. The VU University Medical Center medical ethics committee approved the study.

### Statistics

All data analyses were performed using SPSS statistics 26. To determine the correlation between total IQ and educational achievement, the square root of the Nagelkerke R square was obtained. Furthermore, binary logistic regression analyses were performed. The independent, continuous variables were total, verbal and performance IQ, the dependent binary variable was educational achievement. Gender was examined as a possible effect modifier. Independent *t-*tests and Chi-square tests identified if the variables internalizing problems, externalizing problems, living situation of the adolescent, family functioning, age at which the adolescent started PS/GAHT and age at which the educational achievement was evaluated, were associated with the outcome variable. Since externalizing problems and the age at which the adolescent started GAHT were significantly associated with the outcome variable, these were included and reported as control variable in the logistics regression analyses.

## Results

**Figure 1.**
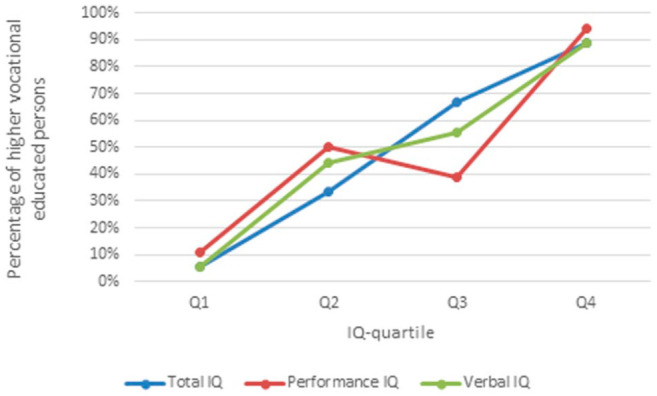
Percentage of higher vocational educated persons per IQ quartile. Quartiles: TIQ: Q1 = 71–89. Q2 = 90–98. Q3 = 98–110. Q4 = 110–136. PIQ: Q1 = 65–89. Q2 = 90–99. Q3 = 100–110. Q4 = 110–135. VIQ: Q1 = 72–88. Q2 = 88–97. Q3 = 97–107. Q4 = 108–136. TIQ = total IQ, PIQ = performance IQ, VIQ = verbal IQ.

The mean total IQ of the participants was 100.29 (SD = 15.07). For verbal IQ, the mean was 99.53 (SD = 15.01), for performance IQ the mean was 100.72 (SD = 14.26). Of the 72 adolescents, 37 were vocational educated (51.4%) and 35 were higher vocational educated (48.6%). The IQ scores and educational achievement were not significantly different between trans men and trans women. All variables were normally distributed. As shown in Figure 1, the associations between total IQ, verbal IQ and educational achievement were linear and therefore suitable for logistic regression. Performance IQ was not entirely linear but sufficient for logistic regression.

The correlation coefficient (Nagelkerke R) between total IQ and educational achievement was 0.71. The binary logistic regression analyses found that for each increase of one point in total IQ score, the chance of being higher educated increased with 1.170 odds (β 0.157 *p* < 0.001, 95% CI: 1.074–1.275) when controlled for externalizing problems and age at which the adolescent started GAHT. For each increase of one point in verbal and performance IQ score, the chance of being higher educated was 1.164 odds (β 0.152, *p* 0.001, 95% CI: 1.068–1.268) and 1.127 odds (β 0.120, *p* < 0.001, 95% CI: 1.054–1.206) respectively when controlled for externalizing problems and age at which the adolescent started GAHT. Gender was not found to be an effect modifier in the association between total, verbal, performance IQ and educational achievement.

## Discussion

The current study on the association between pre-treatment IQ and educational achievement after gender-affirming treatment in transgender adolescents found a strong correlation (Nagelkerke R = 0.71) between pre-treatment IQ and post-treatment (PS, GAHT and gender-affirming surgery) educational achievement after a mean duration of 7.6 years. The association was linear, for each increase of one point in total IQ, the chance of being higher educated increased with 1.170 odds.

The positive correlation between pre-treatment IQ (mean age 12.78 years) and post-treatment educational achievement (mean age 20.40 years) that was found in our sample seems similar to the correlation between IQ at young age and educational achievement later in life in the general population. A meta-analysis that included results from 20 studies with 26,504 participants with an average age of less than 19 years at testing intelligence and an age of over 29 years at the measurement of education, found a correlation of 0.49 (Strenze, 2007). The correlation found in the aforementioned study on the relationship between IQ measured at the age of 11 years and educational achievement measured at the age of 16 years in more than 70,000 English children was even higher: 0.81 (Deary et al., 2007). The comparability of the correlation of IQ and educational achievement between transgender young adults who had received medical affirmative treatment including PS followed by GAHT and the general population thus may suggest that the treatment with PS followed by GAHT in transgender adolescents has not (conspicuously) affected the relation between their IQ and their educational achievement.

Another finding from this study that may suggest that treatment with PS followed by GAHT in transgender adolescents did not noticeably influence the relationship between their cognitive ability and their educational achievement is the fact that the mean of respectively total IQ, verbal IQ and performance IQ of the participating adolescents is almost similar to the general Dutch population according to Statistics Netherlands (Centraal Bureau voor de Statistiek, 2019) whereas their educational achievement was on average higher. In our study, 51.4% of the participants was higher educated compared to 35.5% in the general Dutch population of the age of 15–25 years (Centraal Bureau voor de Statistiek, 2019). One explanation could be that the transgender adolescents in this study were positively stimulated by the psychological counselling they received, so they ended up with more intrinsic motivation to achieve their (educational) goals than cisgender adolescents. After all, these adolescents received psychological support as part of their gender-affirming treatment trajectory for years and their psychosocial and school development was regularly evaluated and when necessary support was organized.

Limitations in this study were the lack of a control group, the small sample size (*N* = 72) and the heterogeneous study population (e.g. age, treatment duration). In addition, since the demographic characteristics of our sample and the methods used to examine IQ and educational achievement were not similar to the studies that have examined this association in the general population, the comparison of the results of these studies should be interpreted with caution. Furthermore, the fact that the adolescents included in this study were on average younger (mean age 13.77 years) when they started PS than adolescents who did not participate (mean age 14.43 years), raises the question of whether these results also apply to adolescents who begin this treatment at an older age (e.g. above the age of 14 or 15 years). In addition, this study had only two measurement points and could therefore not differentiate effects from either PS or GAHT alone. Furthermore, all participants in this study had gender-affirming surgery so the results may not be representative for people who chose not to have gender-affirming surgery. Finally, neurocognitive skills that develop specifically during adolescence like social-emotional processing, executive functioning, risk and reward processing, were not measured in the current study (Chen et al., 2020).

Future studies should use a greater sample size, make use of an age-matched control group and take a longer follow-up including more frequent measurements of IQ and examining effects on executive and neurocognitive functioning (Chen et al., 2020). To investigate which effects are related to which part of treatment, studies focussing specifically on PS, similar studies focussing on only GAHT and studies focussing on the combination of both should be conducted. Future studies should therefore use more time points (at least three) and use measures that better capture neurodevelopmental effects of PS and GAHT on neurocognitive development during adolescence, for example, regarding social-emotional processing, executive functioning, risk and reward processing, possibly including MRI studies (Chen et al., 2020).

## Conclusion

In transgender young adults starting early treatment in adolescence with PS and subsequent GAHT and affirming surgeries, the correlation between pre-treatment IQ at young age and post-treatment educational achievement in young adulthood found in this study seems to be comparable to the general population. Although further research is indicated to clarify the exact effects of treatment with PS and GAHT on neurodevelopment, these results are reassuring in the sense that gender-affirming medical treatment including PS does not seem to negatively affect the association between IQ and educational achievement.
